# Supplementary material for: Tead1a Initiates Transcriptional Priming Through the TEAD1a/YAP‐Notch1‐Spi1/Cebpα Axis to Promote Neutrophil Fate
Source: Adv Sci (Weinh). 2025 Aug 28;12(41):e05441. doi: 10.1002/advs.202505441 (PMC12591127; doi:10.1002/advs.202505441)
Supplement: Supplementary file 4 — Supplementary Dataset [file ADVS-12-e05441-s004.zip › 3-7.zip!/3-7/02-7.cluster/cluster19/Enrich/ego_BP.pdf]

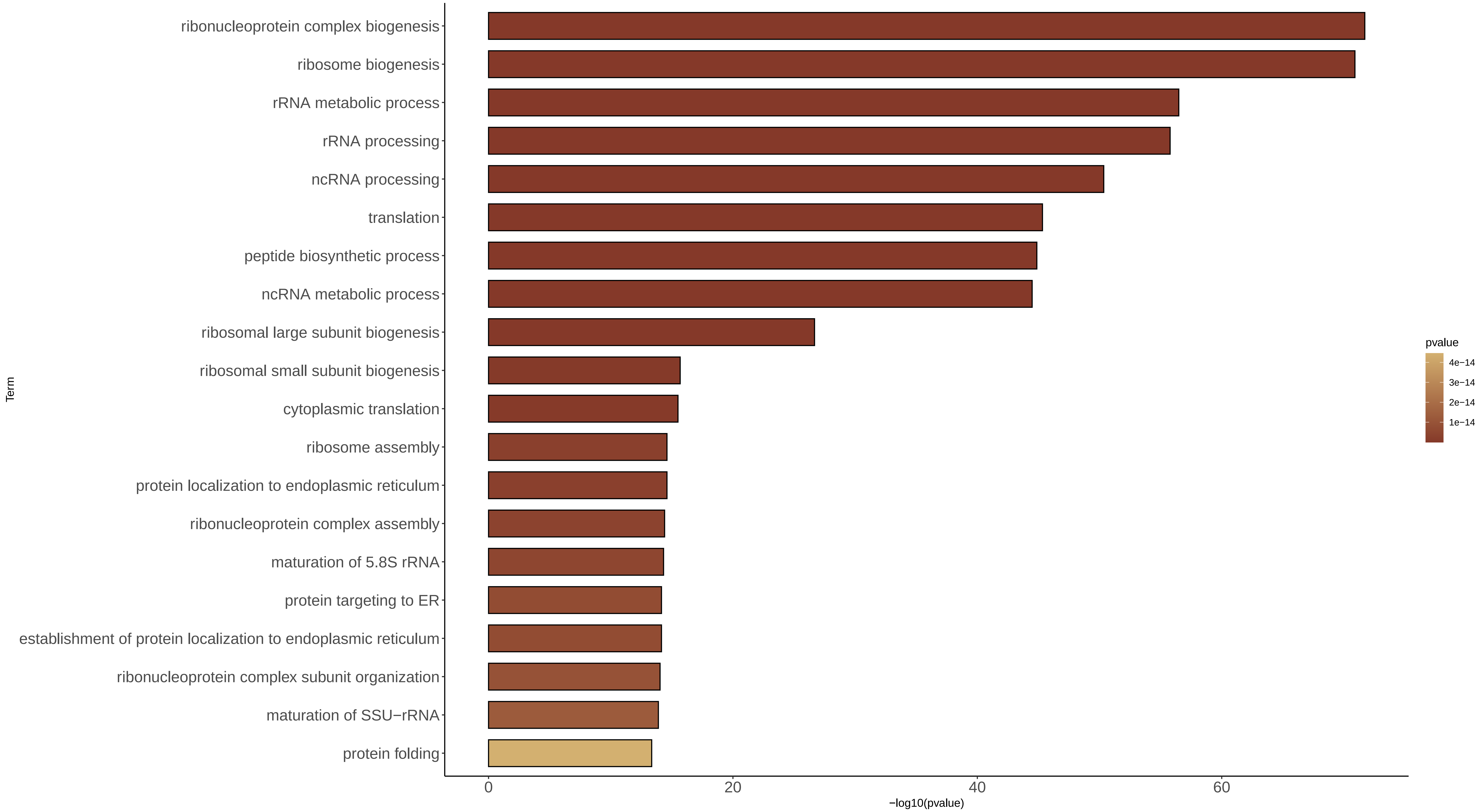
ribonucleoprotein complex biogenesis
ribosome biogenesis
rRNA metabolic process
rRNA processing
ncRNA processing
translation
peptide biosynthetic process
ncRNA metabolic process
ribosomal large subunit biogenesis
ribosomal small subunit biogenesis
cytoplasmic translation
ribosome assembly
protein localization to endoplasmic reticulum
ribonucleoprotein complex assembly
maturation of 5.8S rRNA
protein targeting to ER
establishment of protein localization to endoplasmic reticulum
ribonucleoprotein complex subunit organization
maturation of SSU−rRNA
protein folding
Term
0
20
40
60
−log10(pvalue)
pvalue
4e−14
3e−14
2e−14
1e−14
